# Physical and chemical assessment of 1,3 Propanediol as a potential substitute of propylene glycol in refill liquid for electronic cigarettes

**DOI:** 10.1038/s41598-018-29066-6

**Published:** 2018-07-16

**Authors:** Philippe Bertrand, Vincent Bonnarme, Antoine Piccirilli, Philippe Ayrault, Laurent Lemée, Gilles Frapper, Jérémie Pourchez

**Affiliations:** 1 Institut de Chimie des Milieux et Matériaux de Poitiers, UMR CNRS 7285, 4 rue Michel Brunet, TSA 51106, 86073 Poitiers cedex 09, France; 2 Laboratoires Cérès, 18 chemin de Tison, 86 000 Poitiers, France; 3 Mines Saint-Etienne, Univ Lyon, Univ Jean Monnet, INSERM, U 1059 Sainbiose, Centre CIS, F - 42023 Saint-Etienne, France

## Abstract

Electronic cigarette has the potential to serve as a tobacco cessation aid if the prerequisites which are safety and efficacy in term of nicotine delivery are achieved. The nicotine-based liquids are mainly composed by propylene glycol and glycerol playing the important role of airborne carriers. 1,3 propanediol is proposed as a propylene glycol substitute to potentially improve the thermal stability, nicotine delivery and to decrease inhaled flavors concentrations. We have implemented various thermal, physicochemical and computational methods to evaluate the use of 1,3 propanediol as a substitute (or additional ingredient) to propylene glycol in e-liquids compositions. Our results indicate that 1,3 propanediol is stable upon heating when electronic cigarette are used in recommended conditions. We demonstrate that 1,3 propanediol gave better thermic profile compared to propylene glycol and glycerol, showing less thermal decomposition by-products. In addition, 1,3 propanediol gives to nicotine a more basic environment ensuring a high level of free base nicotine form. We have also established a quantum mechanical based computational method to validate e-liquids as flavor enhancer. Our findings showed that globally 1,3 propanediol seems to have better flavoring properties than glycerol and propylene glycol. Finally, 1,3 propanediol seems to induce quite similar aerodynamic properties compared to propylene glycol and glycerol.

## Introduction

According to the World Health Organization (WHO), the tobacco epidemic is one of the biggest public health threats the world has ever faced^1–3^. Tobacco kills up to half of its users and kills around 6 million people each year. In the scope of tobacco control policy, offer help to quit tobacco is one of the main WHO’s recommendations^1–3^. Nicotine Replacement Therapies (NRTs) and smoking cessation medications allow to increase the chance of quitting smoking by about 50% compared to someone trying to quit unaided^4,5^. The most well-known smoking cessation medication is varenicline. This drug works by lightly stimulating the nicotine receptors in the brain, which is supposed to both ease the cravings and make smoking tobacco much less enjoyable. NRT is a medically-approved way to take nicotine by means other than tobacco use. The idea behind these products is to allow smokers to get nicotine into their body without having to inhale the toxic substances contained in tobacco smoke. The most common NRTs include skin patches, gums, tablets, lozenges, nasal sprays and buccal inhalers, combined or not with anti-depressors are proposed for tobacco cessation. But in term of effectiveness, the results seem to depend on different factors including tobacco consumption level, cure duration and quality of medical follow-up^5^. Nevertheless, some recent studies indicate a quite poor efficiency of usual NRTs probably due to the fact that they deliver nicotine to the brain less rapidly than from smoking cigarettes^6^. In this context, electronic cigarette (EC) appears as an electronic nicotine delivery system having the potential to generate a substantial public health benefit if there is a switch from smoking to EC use. According to Hajek & coll., the specific profile of nicotine delivery from EC is likely to play a major role in their attractiveness to smokers^7^.

But EC is a relatively new consumer product. Safety and effectiveness for tobacco substitution and/or tobacco cessation are still challenged. These two conditions are essential to ensure a correct acceptation by health authorities, medical practitioners, scientific community and consumers. It is clear that safety and efficiency of EC clearly depend on the quality and the performances of both electronic devices and physicochemical properties of the refill liquid compositions (named e-liquid).

Commercial e-liquids containing nicotine are generally composed by glycols (>75 wt%) including propylene glycol (PG) and glycerol (VG) playing the role of airborne carriers. PG is used for its capacity to solubilize nicotine and flavors, VG for its high hygroscopic properties inducing a significant increase of the density of exhaled aerosol. Commercial e-liquid formulations can contain also ethanol and/or water used as diluents (<20 wt%), nicotine (0–3 wt%) and food flavors (1–10 wt%). PG, VG and some flavoring molecules are heat sensitive. glycols containing geminate hydroxyl functions such as PG and VG can be decomposed in certain use conditions of aerosol generation and can induce the formation of toxic volatile compounds such as epoxides (propylene oxide, glycidol or 2,3-epoxy-1-propanol) and aldehydes (formaldehyde, acetaldehyde, acrolein)^8–10^. The formation of these by-products depends on the thermodynamic features such as the power level of the device, the chemical nature of the e-liquid and the puffing regime. On the other hand, PG has a potential to induce skin and airways irritation even in the exposure conditions induced by the use of EC^11–13^. It is the reason why the most important PG manufacturers do not support the use of propylene glycol in EC, nor in artificial (theatrical) fogs due to possible effects on the eye, nose, throat, and respiratory tract tissues^14^.

Concerning flavor molecules which are an essential part of e-liquids compositions, they belong to the important group of authorized food flavors which represents around 2 500 molecules. Also, the global flavor safety evaluation program conducted by the Joint FAO/WHO Expert Committee on Food Additives (JECFA) evaluates the safety of flavoring substances but solely for their use in human food^15^. Occupational Exposure Limits and EC Occupational exposure limits (OELs) have been established for a small number of flavoring substances. OELs have no relevance to exposure to flavors from the use of EC. In this context, toxicologists recommend to use higher toxicological thresholds of concern for flavor ingredients (*e.g*. 170 or 980 µg/day) than for contaminants assessment (*e.g*. 1.5 µg/day)^16^. Moreover, some flavoring molecules can present intrinsic toxicity and can be submitted in certain conditions of use to potential reaction and thermal breakdown inducing the formation of toxic analytes in EC resulting aerosol^17–19^. Strict selection of flavor molecules in terms of potential airways toxicity and thermal stability combined with a limitation of flavors concentration in compositions appears to be a rational and reasonable approach.

In this context, 1,3-propanediol (PDO) is a linear aliphatic diol, which makes it a useful chemical building block. PDO is used for a variety of applications including polymers, personal care products, solvents, and lubricants^20^. PDO is also authorized in foods as flavors carrier and as an alternate to PG. PDO is GRAS certified (“Generally Recognized As Safe”) by FDA^21^. In a recent inhalation toxicity study, PDO tested at 1800 mg/m^3^, PDO does not appear to pose a significant hazard via inhalation of either the gas phase or a gas/aerosol mixture^17^. In cosmetics, 1,3-propanediol is recognized and used as a non-irritant alternative to PG^22^. The objective of this work is to evaluate the chemical and physical properties of PDO and the resulting aerosol features for an intended use in EC. Particularly, thermal stability, aerosol particle size distribution, nicotine stabilization and PDO-flavor molecular interactions were examined.

## Methods

### Materials

1,3 propanediol, propylene glycol, glycerol are supplied by Sigma-Aldrich. Tobacco nicotine compliant to the USP monograph is supplied by Alchem company.

### Differential thermal analysis (DTA-TGA)

The thermal analysis are carried out with Q 600 apparatus (TA Instrument) in the following conditions: air atmosphere at 100 mL/min; temperature range from 25 to 350 °C with a rate of 10 °C/min; 50 mg of sample in an open crucible; alumina crucible as reference material.

### Proton Nuclear Magnetic Resonance (^1^H-NMR)

^1^H NMR spectra were recorded in 5 mm diameter tubes with a Bruker spectrometer (400 MHz), in CDCl_3_. DMSO D6 or D_2_O at 25 °C. The chemical shift scale expressed in ppm was calibrated on the basis of the deuterated solvent or tetramethylsilane as reference. Samples were prepared with 1.8% weight of nicotine in the two selected e-liquids: reference a) propane 1,2 diol (PG, 531 mg), glycerin (VG, 399.3) and water (48,2 mg) and b) propane 1,3-diol (PDO, 1418,9 mg).

### Analytical pyrolysis (Py-GC/MS)

The sample was placed on a quartz wool inserted inside a 2 internal diameter (i.d.)X 40 mm quartz tube. The tube were placed in a CDS Pyroprobe 5150 pyrolyzer directly coupled to a Thermo Trace Ultra gas chromatograph (GC) and a quadrupolar DSQ II mass spectrometer (MS). The resistively heated coil of the apparatus heat the samples with a heating rate of 5 °C.ms^−1^ from room temperature to the selected temperature which was maintained 30 s. The pyrolysis products were sent directly to the GC/MS in a stream of helium. Direct coupling prevents the loss of volatile compounds or possible degradation of the pyrolysis products. The GC separations were conducted in a fused silica capillary column (BPX 5 (SGE), 5% Phenyl Polysilylphenyl-siloxane, 30 m length, 0.25 mm i.d., 0.25 µm film thickness) and helium 5.5 (Messer), 99.9995% purity as carrier gas. The injector was set to 250 °C with a split of 100/1. The column temperature was programmed from 60 to 300 °C at 5 °C.min^−1^ and held at 300 °C for 15 min. The ionization mode was electron impact (70 eV), the data were recorded in full scan mode, the source temperature was 220 °C and the transfer line was set to 280 °C. The pyrolysis or thermochemolysis products were identified on the basis of their GC retention times and by comparison of their mass spectra with analytical standards and library data (NIST).

### Determination of glycols-flavors interactions by a computational method

All molecular species were optimized by density functional theory (DFT) calculations at the M06-2X level^23^. A double-zeta basis set was employed for all atoms, which was increased using polarized functions, 6–31 G(d,p). For each structure, the analytic Hessian was calculated to obtain the vibrational frequencies and to characterize the nature of the stationary point (local minimum). Unscaled frequencies were used to determine the zero-point energy (ZPE) and thermodynamic corrections at 298.15 K. The solvation Gibbs free energies (ΔG_solv_) were computed using the continuum solvation model (SMD) based on the quantum charge density of a solute molecule with a continuum description of the solvent^24^. The SMD model was used with the Barone and Cossi’s implementation of the polarizable continuum conductor-like solvent model (CPCM)^25^, which is based on the polarized continuum model of Tomasi and co-wokers^26^. The SMD-CPCM calculations were performed as M06-2X/aug-cc-pVTZ single point energy calculations on the M06-2X/6–31 G(d,p) geometries. Calculations were performed using the Gaussian 09 computational programs^27^.

### Aerodynamic features

A high-power ENDS was used. This ENDS model was made up of a variable lithium-ion battery (iStick 30 W, Eleaf) and an atomizer (GS Air, Eleaf). Under the support of 2200 mAh battery capacity, the battery gives performance between 5W-30W. The atomizer emphasizes a dual-coil atomizer head, a power in the 8W-20W range and a resistance of 1.5 ohm. Prior to performing particle size experiments, batteries were fully charged, the maximum air inflow position was fixed, and the value of the electrical resistance was checked at 1.5 ± 1 ohm. It is important to underline that PDO based e-liquid used for this study were nicotine-free and flavors-free in order to only exhibit the impact of the PDO solvent on aerodynamic features. To compare to PDO resulting aerosol, two different compositions of refill liquid were used corresponding to 80 wt% PG with 20 wt% VG (noted 80PG/20VG) and 20 wt% PG with 80 wt% VG (noted 20PG/80VG). These formulations were prepared in the laboratory from commercial solutions available on the market to do oneself the refill liquid (100-VG and 100-PG base, A&L, France). Aerosol particle sizing was defined in terms of Mass Median Aerodynamic Diameter (MMAD). The DLPI set-up was used (Dekati Low-Pressure Impactor; Dekati Ltd, Finland) to quantify the mass distribution and MMAD. The protocol was previously described in details^28–30^. An in-house interface was designed to introduce reproducibly a well-controlled volume and duration puff into the inlet of the impactor. Our interface was composed of a 3L-syringe (*Hans-Rudolph, USA*) connected to both the ENDS and the DLPI cascade impactor. Aerosol sampling was carried out considering a 4-s puff.

## Results

### Thermal stability

**Figure 1:**
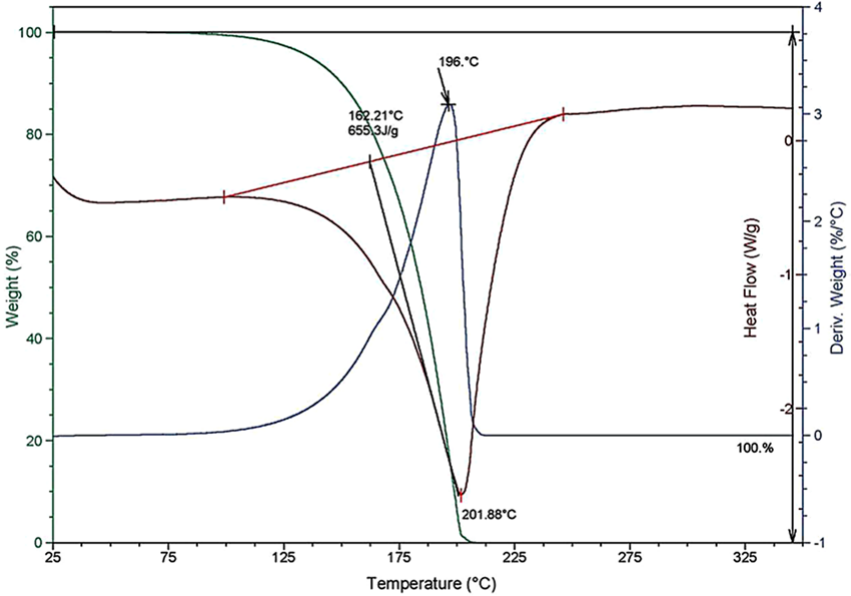
DTA/TGA profiles of the formulation nicotine (2.1 wt%) in PDO.

The behavior of the isolated compounds was first determined by differential thermal analysis (Table S2, supplementary information). Nicotine, PDO and PG are vaporized without any thermal decomposition (absence of exothermic peak), with PG slightly more vaporizable than PDO. VG exhibits a peak of decomposition at 300 °C and a residue of vaporization of 0.2 wt%. Nicotine presents a little residue after vaporization of 0.5 wt%. Then, we investigated the behavior of nicotine-PDO formulation. A co-vaporization of nicotine and PDO is observed without any exothermic peak (Fig. 1).

The thermal stability of formulations of nicotine in PG-VG and PDO-VG were then compared by differential thermal analysis (Table 1, Figure S1 supplementary information). A significant exothermic peak at around 300 °C corresponding to a vaporization residue of 3 wt% is characteristic of the blends PG-VG (c and d) and corresponds to an energy release of 37–38 J/g indicating chemical interactions between PG and VG at high temperature. In contrast, the PDO-VG formulation has no exothermic peak. This observation leads to indicate the absence of any chemical interaction between PDO and VG.

**Table 1:** DTA/TGA Analysis of compositions of PDO, PG, VG and nicotine.

	Endothermal pics				Exothermal pic		
	T_1endo_Max (°C)	T_2endo_Max (°C)	Enthalpy of vaporization (J/g)	Weight lost (%)	T_exo_Max (°C)	Enthalpy of vaporization (J /g)	Weight lost (%)
PDO-VG 60/40 wt%	192	235	704	99.41	—	—	0.32
nicotine-PDO- VG 10/55/35 wt% -VG 35 wt%	187	236	578	98.99	307	−7	0.61
PG-VG 60/40 wt%	162	232	602	97.44	303	−35	1.19
nicotine-PG-VG 10/55/35 wt%- VG 35 wt%	164	223	516	96.87	305	−38	1.41

**Figure 2:**
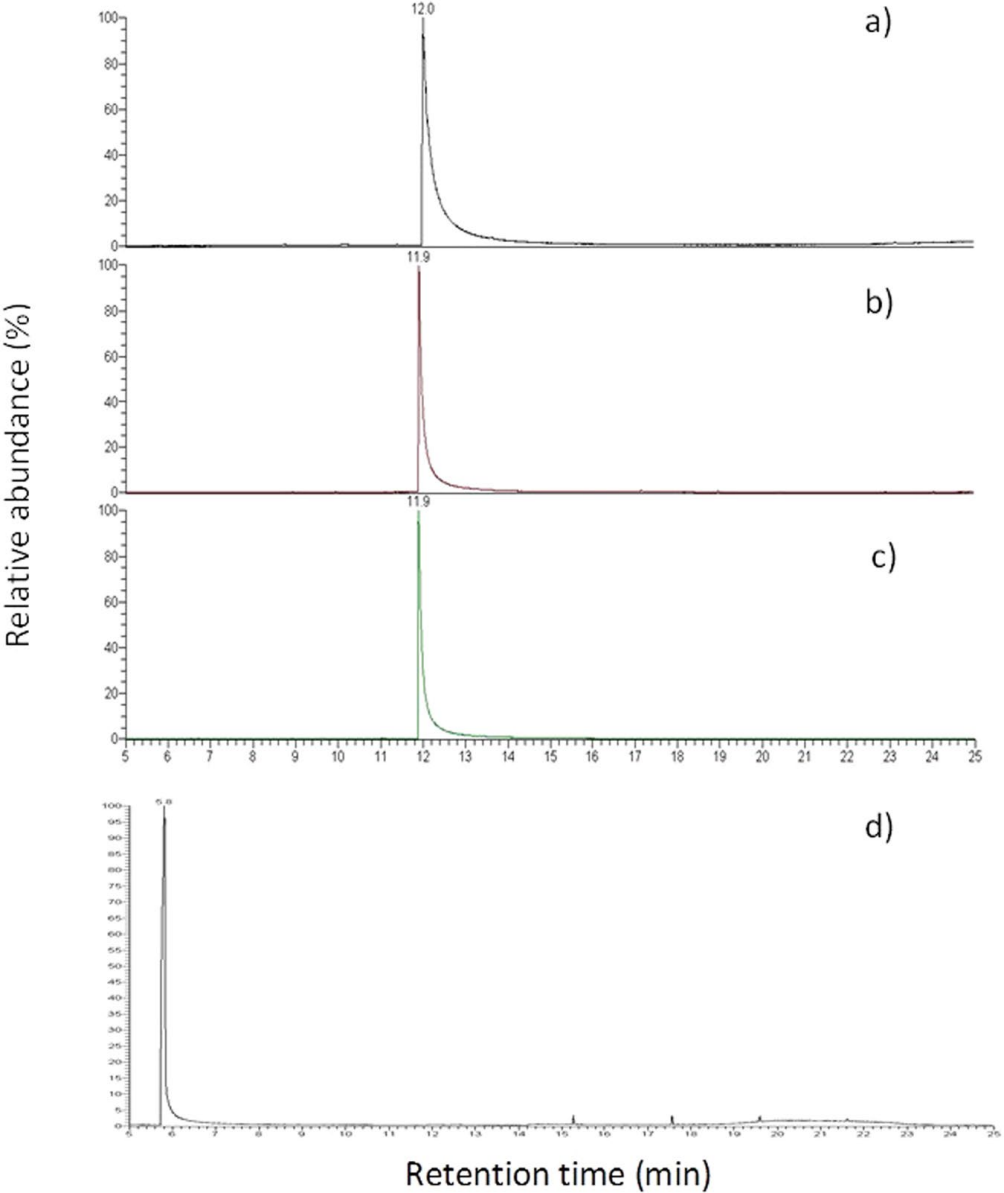
Pyrolysis profiles of nicotine at (**a**) 250 °C for 30 s; (**b**) 400 °C for 30 s; (**c**) 900 °C for 30 s; (**d**) PDO at 250 °C for 30 s.

We have also performed pyrolysis coupled to gas chromatography and mass spectrometry (Py-GCMS) to detect the species produced by heating. Py-GCMS is a powerful tool to determine the stability of molecules trapped in a matrix at moderate to very high temperatures^31–37^, whatever this matrix is, as only volatile compounds at the selected experimental temperature are analyzed. The intrinsic thermal stability of PDO in normal EC conditions (250 °C) with an inert gas flow as the vector (nitrogen) was determined, and for nicotine at different temperatures, regarding its presence in conventional cigarettes. With 30 seconds experiment at the given temperatures (Fig. 2) we found that PDO appeared stable at 250 °C and nicotine is a very stable compound in a heated inert atmosphere. For nicotine, more extreme conditions (heating 60 seconds above 900 °C, Figure S2, supplementary information) lead to the formation of the first side products, a major one being the aromatized nicotyrine.

### Solvent properties and effect on nicotine solubilization

We propose a general ^1^H NMR experiment where various buffered solutions at pH ranging from 1 to 10 were used to establish a reference curve. Upon the first protonation of the pyrrolidine the surrounding hydrogen atoms should have modified chemical shifts, whereas upon the second protonation of the pyridine mostly the hydrogen atoms of this ring should show modified chemical shifts. The chemical shift of all the hydrogen atoms of nicotine was determined in DMSO D_6_ and D_2_O to compare the solvating effects (Table S3 and Figure S3, supplementary information). Additional experiments confirmed the signal identity (2D COSY, Figure S4, supplementary information). Nicotine was diluted at 0.5 mg/ mL in DMSO or D_2_O. 0.2 mL of the buffered solutions were added to 0.5 mL of these nicotine solutions (final nicotine amount 0.5 mg for 0.7 mL). Below this concentration the nicotine signals were difficult to identify. DMSO was not found suitable as the solvation effect was two strong, giving an almost linear response whatever the pH. In contrast, in D_2_O, two gaps were identified that corresponded to the two protonation steps (Figure S3, supplementary information). Stock solutions were then prepared with nicotine at 1.8% weight (18.4 mg) in a mixture of PG + VG + water (531 + 399.3 + 48.2 mg) corresponding to a standard e-liquid composition, compared to a composition with PDO (1418.9 mg) and nicotine (28 mg). The measured chemical shifts are reported in Table 2, clearly demonstrating that in our experimental conditions the PG + VG e-liquids corresponded to pH 8 whereas PDO generates a more basic environment, equivalent to pH 10.

**Table 2:** Chemical shifts of nicotine in e-liquids measured in D_2_O.

	Ha	Hb	Hc	CH_3_	Hd	He	Hf	Hg
PDO	3,18	3,02	2,26	2,00	8,34	8,34	7,73	7,33
PDO + VG + H_2_O	3,28	3,12	2,37	2,09	8,43	8,40	7,81	7,42
Nicotine 1,8 wt % formulation in the buffered conditions								
	Ha	Hb	Hc	CH_3_	Hd	He	Hf	Hg
pH 7	3,65	3,29	2,66	2,26	8,42	8,39	7,80	7,38
pH 8	3,39	3,14	2,44	2,11	8,37	8,35	7,75	7,35
pH 10	3,17	3,02	2,26	1,99	8,34	8,31	7,72	7,33

### Solvent properties and effect on flavor molecules

**Figure 3:**
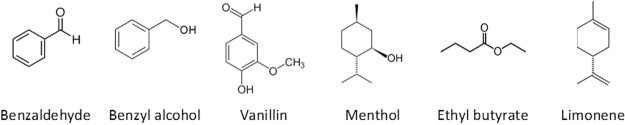
Main flavoring substances used in e-liquids formulations.

We try in this study to characterize the behavior of each glycol in the presence of the main flavoring substances used in e-liquids (Fig. 3). The mathematical descriptors can be related to the solubility with the free energy of solvation, G_solv_, computed using *ab initio* quantum mechanical based approaches combined with implicit solvation models. We present our results based on the polarizable continuum conductor-like solvent model, a particularly efficient way to predict such free energies of solvation. This approach incorporates electronic group effects such as inductive and mesomeric influences on the polarity as well as intramolecular interactions such as hydrogen bonding. These models have been applied in drug design, fragrance property prediction, and atmospheric and soil pollutant partitioning^38^.

Once the ground-state conformers are optimized, the implicit solvation model uses quantum chemically generated charge density surfaces to describe each molecule and its interactions with the chosen solvent. Six flavoring molecules used in the industry were selected (Fig. 3) to validate separately the flavor enhancing properties of PG, VG and PDO. We compared the solubility behavior of each solvent to a given flavoring molecule, and the calculated G_solv._ were normalized, 100 being set arbitrary for all the PDO couples (Table 3). Values below 100 indicate lower flavoring properties. In all cases PDO gave higher flavor enhancement compared to VG. PG was superior to PDO only for the terpenes limonene and menthol. VG being known to reduce flavor in e-liquids, it was not surprising to obtain relative values below 100.

**Table 3:** Relative solvation free energies for the selected couple of e-liquids and flavors, normalize to 100 for PDO.

	Benzaldehyde	Benzyl alcohol	Vanillin	Menthol	Ethyl butyrate	Limonene
PDO	100	100	100	100	100	100
PG	92	88	85	102	87	108
VG	83	79	76	93	75	91

### Aerodynamic features of PDO-based aerosols

**Figure 4:**
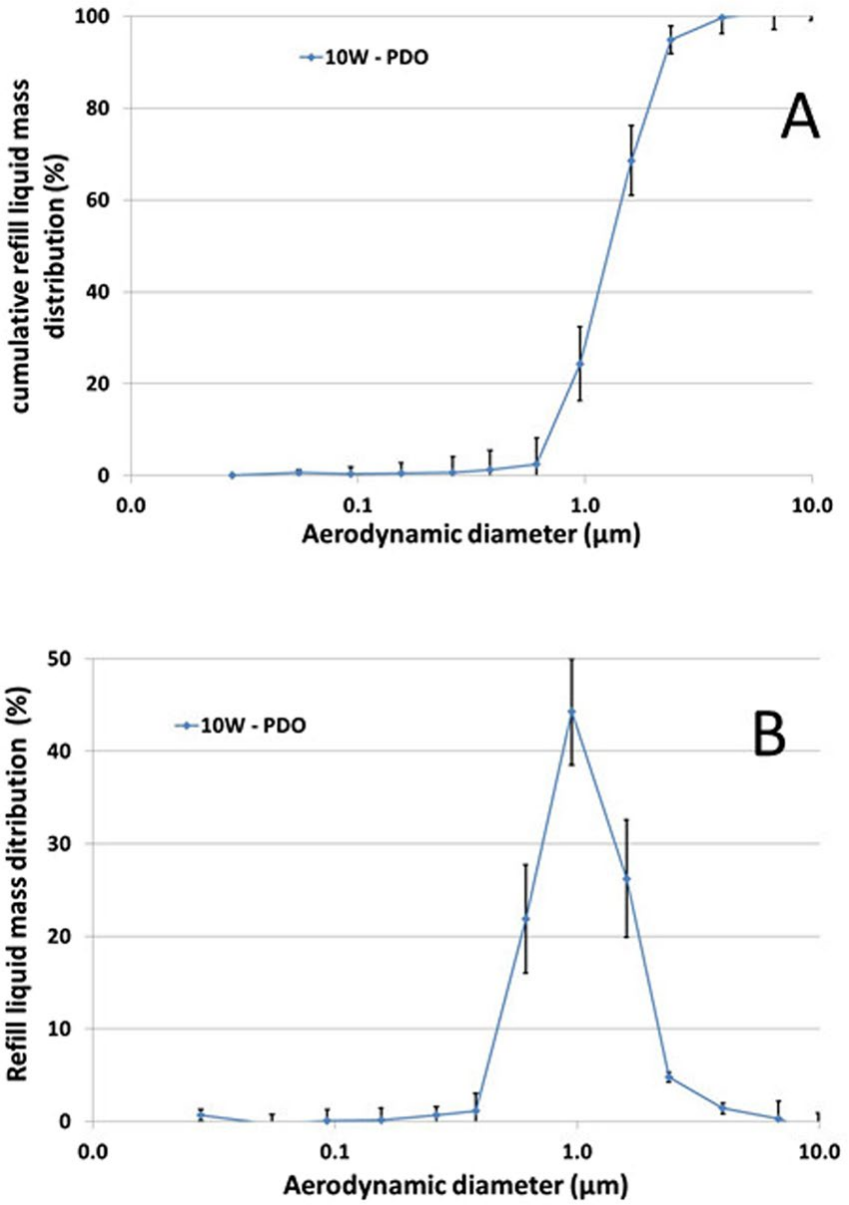
DLPI Impactor-collected data. (**A**) Cumulative mass distribution for the PDO formulation with a power level of EC fixed at 10 W. Experiments performed in triplicate. (**B**) Frequency mass distribution for the PDO formulation with a power level of EC fixed at 10 W. Experiments performed in triplicate.

**Figure 5:**
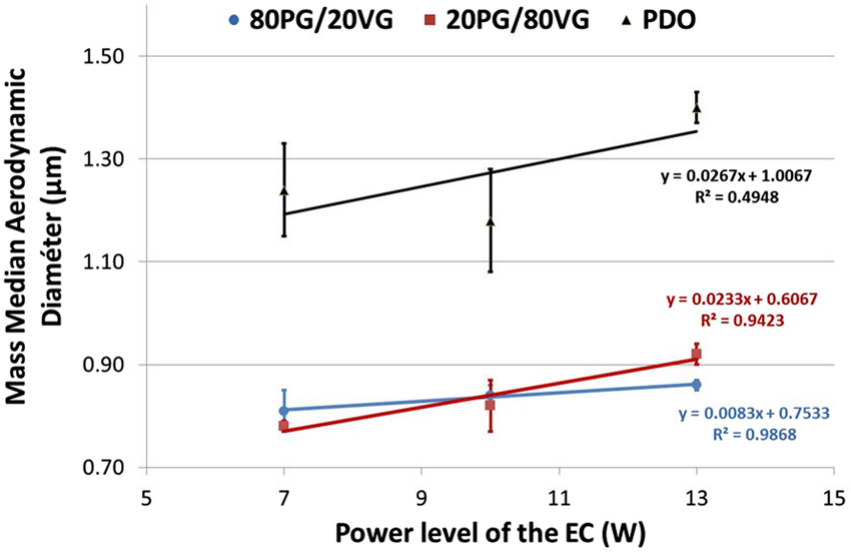
Impact of the power level of the EC on the Mass Median Aerodynamic Diameter (MMAD). Experiments performed in triplicate.

**Figure 6:**
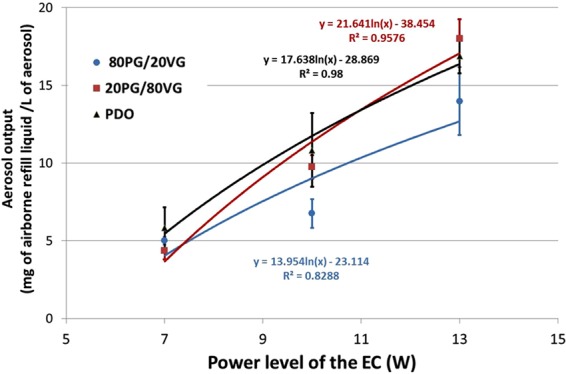
Impact of the power level of the EC on the aerosol output. Experiments performed.

Figure 4 showed impactor-collected data by means of frequency mass distribution and cumulative mass distribution. Findings present a dominant mode at 949 nm and a MMAD of 1.33 ± 0.09 µm at 10 W (Fig. 4). Besides the impact of the power level on the aerodynamic features was also investigated in the 7–13 W range. A slight increase of the MMAD was noticed when the power level rises (Fig. 5). A linear correlation highlighting the rise of MMAD as a function of the power level was clearly put in evidence all e-liquid compositions (80PG/ VG, 20PG/ VG, PDO). By contrast, PDO e-liquid seems to induce higher MMAD compared to PG + VG based formulation. Finally, Fig. 6 presents the aerosol dose emitted as a function of the power level of the EC ranging from 7 W to 13 W. Unsurprisingly the power level appeared as a very important parameter playing on the aerosol output generated by EC. A logarithmic law seems to be quite satisfactory in order to predict the rise of aerosol output when the power of the EC battery increases (Fig. 6). Secondly, the impact of the refill liquid composition showed a similar tendency for all formulations.

## Discussion

Chemical and physical properties of PDO, PG and VG are listed in supplementary information Table S1. In contrast to PG and VG, PDO is not a geminate glycol explaining why PDO has a lower viscosity than PG and VG. In term of volatility, PDO presents higher heat of vaporization and boiling point than PG but its relative volatility remains significantly higher than VG. Regarding pKa values, PDO is significantly less acid than PG and VG and suggest that PDO can be considered like an aprotic solvent. If the 3 glycols present very high water solubility, their different chemical structures and acidic properties could have a significant impact on their respective solvation properties toward organic solutes such as nicotine and flavor substances.

The differential thermal analysis showed that PDO is vaporized without any thermal decomposition. We can notice that PG is slightly more vaporizable than PDO. This result is in good accordance with theoretical data indicating an enthalpy of vaporization of 70 ± 5 kJ/mol (respectively 67 ± 8 kJ/mol) and a boiling point of 214.0 ± 3.0 °C (respectively 187.2 ± 2.0 °C) for the PDO compound (respectively for the PG compound). The thermal behavior of nicotine-PDO formulation clearly exhibited a co-vaporization process of nicotine and PDO, confirming the absence of chemical interaction between nicotine and PDO. This result suggests that PDO is particularly adapted to ensure a constant delivery of nicotine during vaping process. Besides, the blends PDO-VG seems to be more stable than PG-VG ones containing or not nicotine. Firstly, a significant exothermic peak at around 300 °C was noticed, indicating chemical interactions between PG and VG at high temperature. By contrast, the PDO-VG formulation has no exothermic peak and confirms the absence of any chemical interaction between the both glycols, *i.e*. PDO and VG. All things considered, PDO seems to stabilize glycerol at high temperature.

In EC devices, the temperature is below 300 °C, as higher temperatures are considered a misuse. Indeed, e-liquids can be validated for such temperature, but for security reasons it could be of interest to test them in extreme conditions. We have performed pyrolysis coupled to gas chromatography and mass spectrometry (Py-GCMS) to detect the species produced by heating. The intrinsic thermal stability of PDO in normal EC conditions (250 °C) is confirmed by Py-GCMS. Besides, nicotine appears also as a very stable compound in a heated inert atmosphere. Nevertheless, in case of more extreme conditions (heating 60 seconds above 900 °C, Figure S2, supplementary information), nicotine lead the production of by-products such as the aromatized nicotyrine.

The potential of PDO for better delivery of nicotine was then assessed. In tobacco cigarette smoke the free and protonated nicotine forms control the availability of nicotine^39^, a better delivery is obtain for the nicotine free base, the major one in basic environments^40^. This has justified the use of ammonia in cigarettes^41^ but with no effect in EC^42^. The pKas of nicotine are 7.85 for the pyrrolidine and 3.15 for the pyridine. Indeed a e-liquid should have a pH higher than 7.85 to ensure full delivery of free nicotine. Several strategies were proposed to correlate the pH of e-liquids^39^ to the delivery of nicotine or the corresponding vapors with limited usefulness^43^, mostly based on the classical pHmetry. ^1^H Nuclear magnetic resonance (^1^H NMR) is used to identify hydrogen atoms in molecules depending on their environment. The pH of the nicotine environment may results in a single or double nicotine protonation that influences all the hydrogen atoms around the protonated sites, leading to varying chemical shifts. The NMR profiles of cigarette smoke condensate^44^ or in EC device^45^ may lead to complex NMR profiles difficult to globally interpret, although NMR is used to verify cigarette authenticity^46^. The measured chemical shifts by ^1^H NMR clearly showed that the PG + VG e-liquids corresponded to pH 8 whereas PDO generates a more basic environment, equivalent to pH 10. This can be explained by the structure of PG or VG that are both 1,2-diols with hydrogen bonding favored between the two hydroxyl groups allowing easier proton removal and thus more acidity, which is not the case for 1,3 diol PDO. This is also correlated to their known pKa values (PDO = 16.3, PG = 14.8, VG = 13.5). Additional pH measurements by pHmetry confirmed these NMR observations as reported in Table S4, supplementary information, for various composition of PDO, PG or VG. As postulated by Pankow and its coefficient^47^, nicotine 1.8% in PDO is 100% free base.

In e-liquid formulations, PG plays the role of flavors carrier and is recognized as an efficient solvent for the main flavoring molecules whereas VG is used for its vaping properties and its capability to form a thick exhaled aerosol but VG limits the flavors release and requires to increase significantly the concentration of flavoring molecules in formulations and to use specific additives to enhance the sensorial properties (sugars, ethanol, butane 2,3 dione or diacetyle, …). From a safety viewpoint, limiting the flavors concentration and additives in e-liquids can be considered as a key objective. Flavors release depends mainly on the chemical interactions between solvent and flavoring molecules, notably the solvation capability. We try in this study to characterize the behavior of each glycol in the presence of the main flavoring substances used in e-liquids (Fig. 3). As flavors in EC may produce toxic products^18^ and the methods used to characterize flavors in e-liquids^48,49^ are not efficient to rationalize the flavoring effect of a formulation, a validated predictive tool to assess the behavior of flavors in e-liquid is of high importance. The direct perception by the nose or upon inhalation is vapers-dependent and a more rational way would be a numerical simulation measuring the interactions between a e-liquid and a given flavoring molecule. In all cases, the *ab initio* quantum mechanical based approaches showed that PDO has higher flavor enhancement compared to VG. Moreover, PG seems to be superior to PDO only for the terpenes limonene and menthol, an observation that may be further developed. Finally, our findings confirmed that VG significantly reduce flavor in e-liquids. Any blended formulation of PDO, PG or VG may be evaluated based on the percentage of each e-liquid component in the formulation, the same percentage being applied to the ΔG°_solv_ value we obtained for each component, for a given flavor.

^1^H NMR appeared to be a suitable method to determine the pH of a formulation and its impact on the nicotine forms, with a link to the delivery properties. The preliminary numerical experiments open an interesting way for the understanding of the role of solvent as flavor enhancer in e-cigarette. Nevertheless, more work has to be done to model the more complex flavors used in e-cigarette formulation that may contain dozens of molecules, potentially able to interact with each other. Others solvation models are envisaged where i) explicit solvent molecules surround the flavoring molecule, and ii) the dynamical behavior of a liquid upon temperature is taken in account such as in molecular dynamics simulations^50,51^.

Finally, impactor-collected data showed for PDO-based aerosol a dominant mode at 949 nm of aerodynamic diameter as well as a MMAD of 1.33 ± 0.09 µm at 10 W (Fig. 4). Using similar experimental conditions, we observed significant lower MMAD around 0.85 µm of aerodynamic diameter for PG + VG-based aerosol. Besides the impact of the power level on the aerodynamic features was also investigated in the 7–13 W range. A slight increase of the MMAD was noticed when the power level rises. A linear correlation highlighting the rise of MMAD as a function of the power level was clearly put in evidence all e-liquid compositions (80PG/ VG, 20PG/ VG, PDO). By contrast, PDO e-liquid seems to induce higher MMAD compared to PG + VG based formulation. Recently, we deeply investigated the impact of power level and refill liquid composition on the aerosol output and particle size distribution generated by PG + VG-based aerosol^29^. Based on these results, this study supports the conclusion that, globally, similar aerodynamic behavior was observed between PDO-based and PG + VG-based aerosol.

## Conclusion

We have developed complementary methods to characterize PDO as a e-liquid that may be applied to other formulating ingredients such as PG. These methods include thermal stability, pH determination by a rapid and simple method, aerodynamic features and flavor enhancing properties with a numerical predictive model to access to the associated properties such as solubility. To sum-up, our results indicate that:

- PDO is doubtless stable upon heating when EC are used in recommended conditions (250 °C).
- PDO seems to have better thermal behavior showing less thermal decomposition by-products compared to PG and VG.
- PDO seems to give to nicotine a more basic environment ensuring a 100% free base nicotine form, although once in the aerosol it could return to its protonated state.
- PDO shows globally better flavoring properties than VG and PG, except for the case of the two terpenes limonene and menthol.
- PDO seems to induce quite similar aerodynamic properties compared to usual aerosol features generated by the use of PG + VG refill liquid in electronic cigarettes.

## Electronic supplementary material

supplementary information
