# Supplementary material for: Physical and chemical assessment of 1,3 Propanediol as a potential substitute of propylene glycol in refill liquid for electronic cigarettes
Source: Sci Rep. 2018 Jul 16;8:10702. doi: 10.1038/s41598-018-29066-6 (PMC6048019; doi:10.1038/s41598-018-29066-6)
Supplement: Supplementary file 1 — supplementary information [file 41598_2018_29066_MOESM1_ESM.docx]

**Physical and chemical assessment of 1,3 Propanediol as a potential substitute of propylene glycol in refill liquid for electronic cigarettes**

Philippe Bertrand^1*^, Vincent Bonnarme^2^, Antoine Piccirilli^2^, Philippe Ayrault^1^, Laurent Lemée^1^, Gilles Frapper^1^, Jérémie Pourchez^3*^

1 Institut de Chimie des Milieux et Matériaux de Poitiers, UMR CNRS 7285, 4 rue Michel Brunet, TSA 51106, 86073, Poitiers cedex 09, France

2 Laboratoires Cérès, 18 chemin de Tison, 86 000 Poitiers

3 Mines Saint-Etienne, Univ Lyon, Univ Jean Monnet, INSERM, U 1059 Sainbiose, Centre CIS, F - 42023 Saint-Etienne France

### Running title: assessment of 1,3 Propanediol for electronic cigarette

* Corresponding author

Corresponding author: Jérémie POURCHEZ

École Nationale Supérieure des Mines de Saint-Etienne

158 cours Fauriel, CS 62362

42023 Saint-Etienne Cedex 2 FRANCE.

Email address: [pourchez@emse.fr](mailto:pourchez@emse.fr) **-** Telephone number: +33477420180

Corresponding author: Philippe BERTRAND

Institut de Chimie des Milieux et Matériaux de Poitiers

4 rue Michel Brunet, TSA 51106

86073 Poitiers Cedex 9 FRANCE.

Email address: philippe.bertrand@univ-poitiers.fr **-** Telephone number: +33549454085

**Supplementary information**

**Table S1.** Chemical and physical properties of PDO, PG and VG

| Glycol / Physical property | PDO | PG | VG |
| --- | --- | --- | --- |
| Formula | C_3_H_8_O_2_ | C_3_H_8_O_2_ | C_3_H_8_O_3_ |
| Chemical structure | 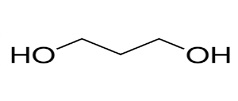 | 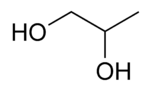 | 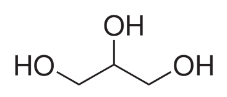 |
| Physical description | Colorless and odorless liquid | Colorless and odorless liquid | Colorless, odorless and syrupy liquid |
| Molecular weight, g.mol^-1^ | 76.095 | 76.095 | 92,094 |
| Boiling point, °C (760 mmHg) | 214 | 187 | 290^1^ |
| Flash point, °C (closed cup) | 129 | 103 | 160 |
| Density | 1,0597 | 1,0361 | 1,2613 |
| Vapor pressure a 25°C, mm Hg | 0.044 | 0,130 | < 10^-3^ |
| Index of refraction at 20°C | 1,439 | 1,433 | 1,4746 |
| Viscosity at 20°C, cP | 52 | 56 | 1412 |
| pKa | 16,3 | 14,8 | 13,5 |
| Heat of vaporization, J/g | 771 | 753 | 853 |
| Water solubility at 25°C, g/l | > 1000 | > 1000 | > 1000 |

**Temperature of decomposition**

Thermal analysis is the analysis of a change in a property of a sample which is related to an imposed change in the temperature. The analysis of the change in the mass of a sample on heating is known as Thermogravimetric analysis (TGA). TGA measures mass changes in a material as a function of temperature under a controlled atmosphere. Its principal uses include measurement of a material's thermal stability and composition. TGA is most useful for dehydration, decomposition, desorption, and oxidation processes. The most widely used thermal method of analysis is Differential thermal analysis (DTA). In DTA, the temperature of a sample is compared with that of an inert reference material during a programmed change of temperature. The temperature should be the same until thermal event occurs, such as melting, decomposition. In an endothermic event takes place within the sample, the temperature of the sample will lag behind that of the reference and a minimum will be observed on the curve. On the contrary, if an exothermal event takes place, then the temperature of the sample will exceed that of the reference and a maximum will be observed on the curve. The area under the endotherm or exotherm is related to the enthalpy of the thermal event, ΔH. In the case of liquid formulations, TGA-DTA modes can be used to determine the enthalpies of vaporization, the thermal and oxidative stability and transformation temperatures.

**Table S2.** TDA/TGA Analysis of PDO, PG, VG and nicotine

| Compound | Endothermic peak (°C) | Enthalpy of vaporization (J/g) | Exothermic peak (°C) | Residue at 350°C (wt%) |
| --- | --- | --- | --- | --- |
| PDO | 188 | 821 | - | - |
| PG | 160 | 757 | - | - |
| VG | 251 | 1127 | - | 0,2 |
| Nicotine | 197 | 175 | 300 | 0,5 |

**Figure S1.** DTA/TGA profiles of VGcols-based formulations: (a) PDO-VG 60/40 wt%, (b) nicotine-PDO-VG 10/55/35 wt%- VG 35 wt%, (c) PG-VG 60/40 wt%, (d) nicotine-PG-VG 10/55/35 wt%- VG 35 wt%


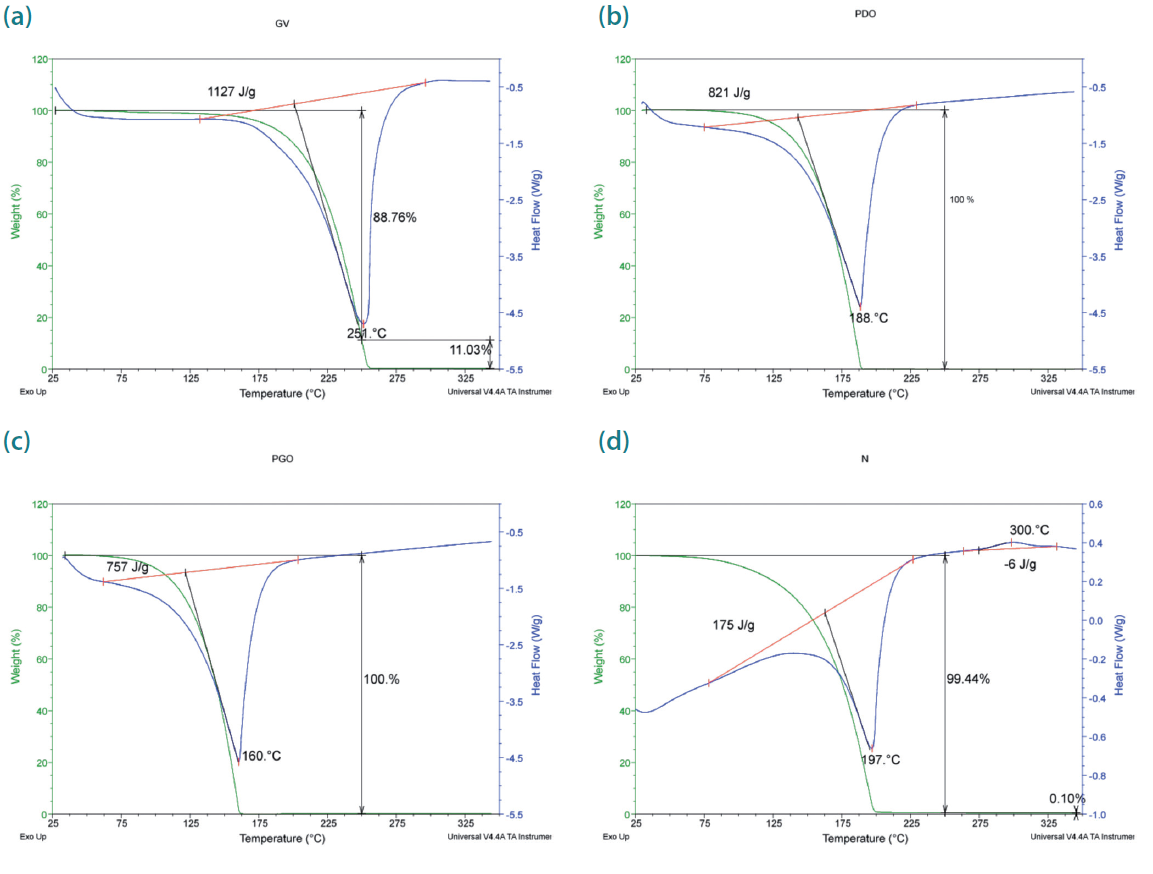


**Figure S2.** Pyrolysis profiles of nicotine at a) 900°C for 60 s; b) 1200°C for 60s; c) 1400°C for 60 s


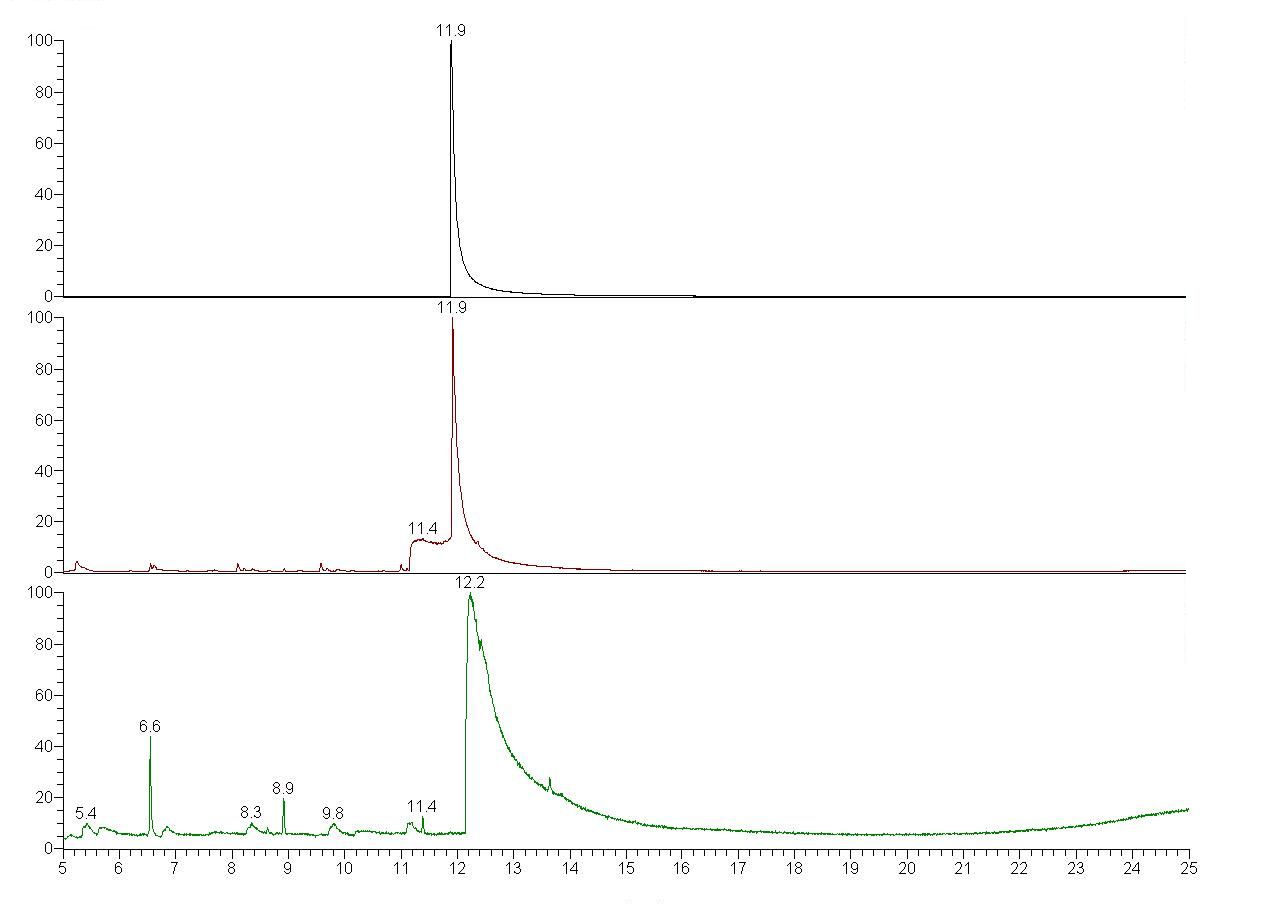


c)

b)

a)

Relative abundance (%)

Retention time (min.)

**Table S3.** ^1^H NMR values of chemical shifts of nicotine measured in DMSO and D_2_O at selected pH with nicotine 1.8 wt % formulation

| DMSO |  |  |  |  |  |  |  |  |
| --- | --- | --- | --- | --- | --- | --- | --- | --- |
| pH | Ha | Hb | Hc | CH_3_ | Hd | He | Hf | Hg |
| 2 | 3,71 | 3,34 | 2,68 | 2,27 | 8,49 | 8,48 | 7,85 | 7,45 |
| 3 | 3,4 | 3,2 | 2,4 | 2,12 | 8,44 | 8,42 | 7,8 | 7,41 |
| 7 | 3,12 | 3,06 | 2,1 | 1,98 | 8,37 | 8,36 | 7,75 | 7,38 |
| 10 | 3,1 | 3,06 | 2,1 | 2.0 | 8,4 | 8,4 | 7,75 | 7,4 |
| D_2_O |  |  |  |  |  |  |  |  |
| pH | Ha | Hb | Hc | CH_3_ | Hd | He | Hf | Hg |
| 1 | 4,5 | 3,82 | 3,28 | 2,76 | 8,91 | 8,8 | 8,67 | 8,08 |
| 2 | 4,36 | 3,75 | 3,23 | 2,68 | 8,55 | 8,52 | 7,95 | 7,49 |
| 3 | 4,4 | 3,73 | 3,22 | 2,67 | 8,54 | 8,51 | 7,92 | 7,48 |
| 5 | 4,35 | 3,71 | 3,23 | 2,65 | 8,53 | 8,5 | 7,91 | 7,46 |
| 6 | 3,61 | 3,27 | 2,62 | 2,23 | 8,42 | 8,39 | 7,8 | 7,38 |
| 7 | 3,65 | 3,29 | 2,66 | 2,26 | 8,42 | 8,39 | 7,8 | 7,38 |
| 8 | 3,39 | 3,14 | 2,44 | 2,11 | 8,37 | 8,35 | 7,75 | 7,35 |
| 10 | 3,17 | 3,02 | 2,26 | 1,99 | 8,34 | 8,31 | 7,72 | 7,33 |

**Figure S3.** Representation of the chemical shifts (δ ppm) of key hydrogens of the nicotine as a function of pH for left: DMSO D6 and right: D_2_O.

|  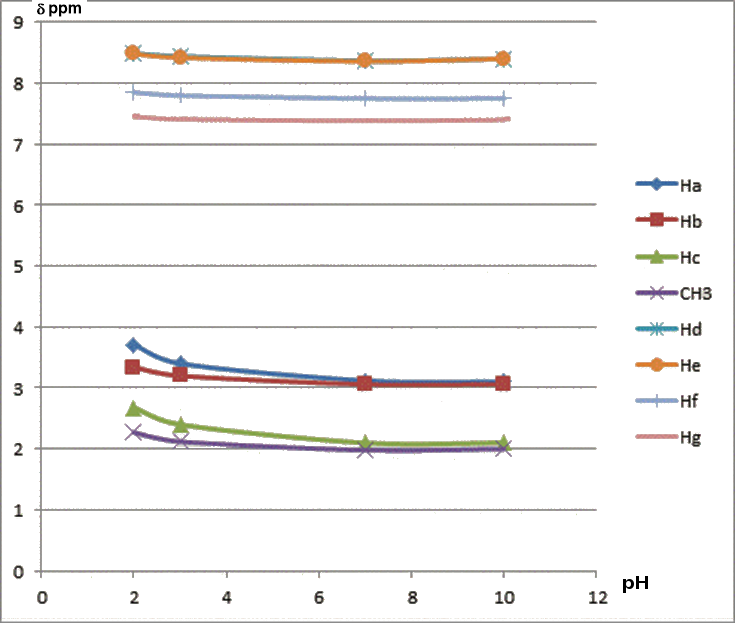 | **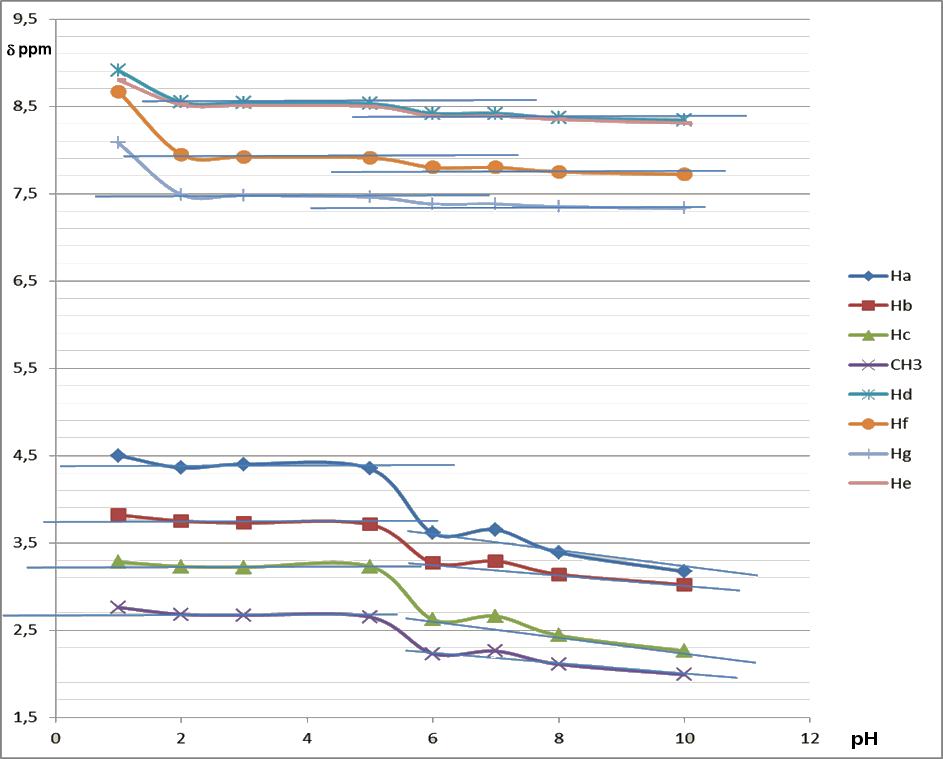** |
| --- | --- |

**Figure S4.** ^1^H NMR 2D COSY of nicotine in buffer pH 5 (left) et zooms for regions in the range 2-2,8 and 3-3,8 ppm (right).

| 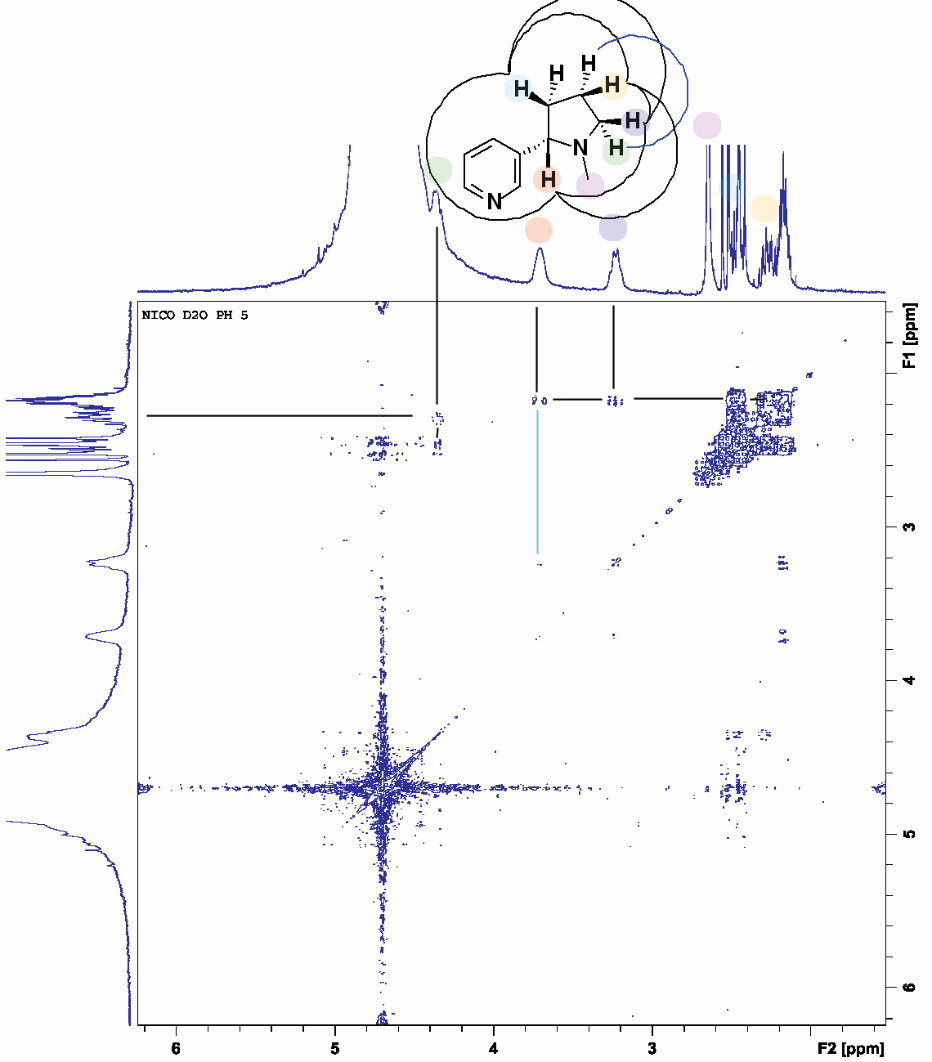 | 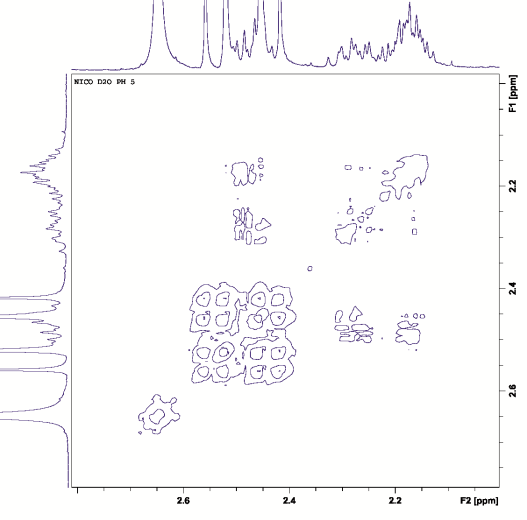  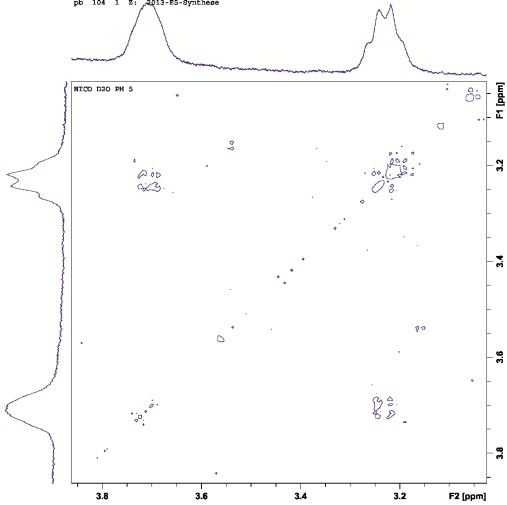 |
| --- | --- |

**Table S4.** pH measurements of glycols formulations

|  | Composition (wt%) | | | pKa (25°C) |
| --- | --- | --- | --- | --- |
| PDO | 100 | 60 | - | 16,3 ^15^ |
| PG | - | - | 60 | 14,8 |
| VG |  | 40 | 40 | 13,5 |
| pH | 9,6 | 7,4 | 6,8 |  |
